# AIRE polymorphism, melanoma antigen-specific T cell immunity, and susceptibility to melanoma

**DOI:** 10.18632/oncotarget.11506

**Published:** 2016-08-22

**Authors:** Giuseppina Conteduca, Daniela Fenoglio, Alessia Parodi, Florinda Battaglia, Francesca Kalli, Simone Negrini, Samuele Tardito, Francesca Ferrera, Annalisa Salis, Enrico Millo, Giuseppe Pasquale, Giusi Barra, Gianluca Damonte, Francesco Indiveri, Soldano Ferrone, Gilberto Filaci

**Affiliations:** ^1^ Centre of Excellence for Biomedical Research, University of Genoa, Genoa, Italy; ^2^ Department of Internal Medicine, University of Genoa, Genoa, Italy; ^3^ IRCCS AOU San Martino – IST, Genoa, Italy; ^4^ Department of Clinical and Experimental Medicine, Second University of Naples, Naples, Italy; ^5^ Department of Surgery, Massachusetts General Hospital, Harvard Medical School, Boston, MA, USA

**Keywords:** AIRE, MAGE, medullary thymic epithelial cells, single nucleotide polymorphism, tolerance, Immunology and Microbiology Section, Immune response, Immunity

## Abstract

AIRE is involved in susceptibility to melanoma perhaps regulating T cell immunity against melanoma antigens (MA). To address this issue, AIRE and MAGEB2 expressions were measured by real time PCR in medullary thymic epithelial cells (mTECs) from two strains of C57BL/6 mice bearing either T or C allelic variant of the rs1800522 AIRE SNP. Moreover, the extent of apoptosis induced by mTECs in MAGEB2-specific T cells and the susceptibility to *in vivo* melanoma B16F10 cell challenge were compared in the two mouse strains.

The C allelic variant, protective in humans against melanoma, induced lower AIRE and MAGEB2 expression in C57BL/6 mouse mTECs than the T allele. Moreover, mTECs expressing the C allelic variant induced lower extent of apoptosis in MAGEB2-specific syngeneic T cells than mTECs bearing the T allelic variant (*p* < 0.05). Vaccination against MAGEB2 induced higher frequency of MAGEB2-specific CTL and exerted higher protective effect against melanoma development in mice bearing the CC AIRE genotype than in those bearing the TT one (*p* < 0.05). These findings show that allelic variants of one AIRE SNP may differentially shape the MA-specific T cell repertoire potentially influencing susceptibility to melanoma.

## INTRODUCTION

AIRE gene, located in the 21q22.3 region, encodes a powerful transcriptional transactivator [1–4]. It is highly expressed in thymus [5] by a subpopulation of medullary thymic epithelial cells (mTECs) [6]. In mTECs AIRE is responsible for the expression of genes known as promiscuously expressed genes encoding tissue-restricted self-antigens (TRAs) [7, 8]. The expression of TRAs in mTECs allows negative selection of auto-reactive lymphocytes having high affinity for the cognate auto-antigen, but not of those with low/intermediate affinity [9]. Hence, potentially auto-reactive T cell clones specific for several auto-antigens, including melanoma antigens (MA) such as MAGE [6, 10, 11], are exported to the periphery.

AIRE gene is polymorphic and its polymorphisms may be associated to disease susceptibility [12–14]. Recently we observed that reciprocal variant alleles of AIRE single nucleotide polymorphisms (SNPs) rs1055311, rs1800520 and rs1800522 are differently present in healthy subjects and in melanoma patients [15]. Interestingly, the protective allelic variants of these SNPs are associated with increased frequency of two MAGE-1-specific T-cell clonotypes in healthy subjects, suggesting that their protective effect may be mediated by selection/expansion of MA-specific T cells. In the present study we have investigated this mechanism, taking advantage of two strains of C57BL/6 mice each of which bears selectively one of the two variant alleles of rs1800522 AIRE SNP. MAGEB2 gene, expressed both by C57BL/6 mice [16] and B16F10 melanoma cells (our observation), was selected as a reference antigen for measuring MA-specific T cell response because MAGE antigens are AIRE-dependent antigens expressed by mTECs [17–21] and have been suggested to be tumor rejection antigens [22, 23]. The results show that the two variant alleles of the rs1800522 AIRE SNP differently modulate MAGEB2-specific T cell survival and *in vivo* susceptibility to melanoma. This finding suggests a role for AIRE polymorphisms in melanoma protection or susceptibility through differential shaping of MA-specific T cell repertoire.

## RESULTS

### Identification of a homologous SNP in human and mouse AIRE gene

**Figure 1:**
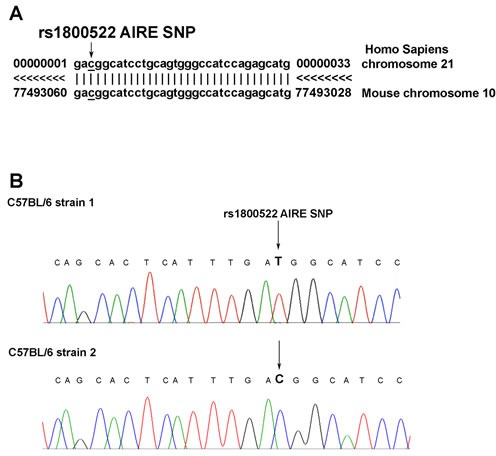
Analysis of AIRE genomic sequences encompassing the rs1800522 SNP in humans and mice **A.** Comparison of human and mouse AIRE gene sequences. Side by side sequence alignment of the region comprised between nucleotides 13207 and 13241 downstream of the translation initiation codon of human AIRE gene on chromosome 21, containing the rs1800522 SNP, and of that comprised between nucleotides 11650 and 11690 of mouse AIRE gene on chromosome 10 shows the identity of the two regions. Data were obtained from UCSC database. **B.** Sequence analysis of the exon 14 of AIRE gene in C57BL/6 mice strain 1 (upper row) and strain 2 (lower row). The analysis was performed on DNA extracted from mTECs of each of the two strains of C57BL/6 mice.

In our recent analysis of AIRE SNPs in patients with melanoma we have found that three SNPs are associated with low risk of melanoma development [15]. To test whether these polymorphisms were present in mouse AIRE gene, we aligned mouse and human AIRE sequences using the UCSC database (www.genome.ucsc.edu/cgi-bin/hgBlat). We found that mouse AIRE gene sequence between 13207 and 13241 base pairs of exon 14 (located in chromosome 10), has a 86% homology with that between 11650 and 11690 base pairs of the corresponding exon of human AIRE gene (located in chromosome 21) (Figure 1A) where rs1800522 SNP is mapped. The possible genotypes of rs1800522 SNP are CC, TT and CT. To test whether this polymorphism was present in the mouse gene, we sequenced exon 14 of mouse AIRE gene in C57BL/6 mice purchased from either Harlan srl or Charles River. Interestingly, we found that mice purchased from Harlan (herein referred to as strain 1) expressed the TT genotype, while those purchased from Charles River (herein referred to as strain 2) expressed the corresponding CC genotype (Figure 1B).

### AIRE regulates MAGEB2 gene expression in mTECs by epigenetic mechanisms

**Figure 2:**
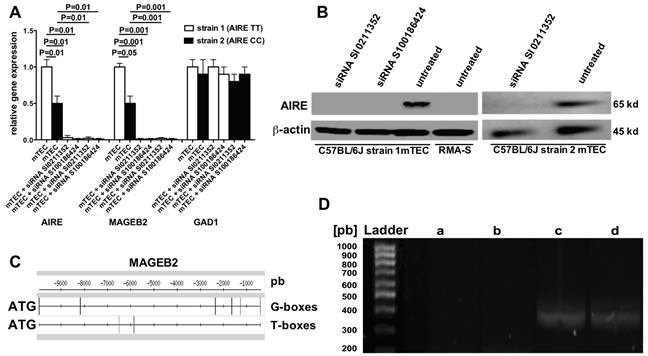
AIRE regulation of MAGEB2 gene expression **A.** Gene expression of AIRE, MAGEB2 and GAD1 in mTECs treated or not with two AIRE-specific siRNA evaluated by real time PCR. White and black columns refer to mTECs extracted from C57BL/6 mice strain 1 and 2, respectively. The results are the mean of three independent experiments. **B.** Western blot analysis of AIRE protein expression in mTECs from both C57BL/6 mice strain 1 and 2 treated or not with AIRE-specific siRNA, and in RMA-S cells, used as a negative control. **C.** Localization of AIRE-binding sites (T-boxes and G-boxes) from 0.5kb to approximately 10 kb upstream to ATG initiation codon of the MAGEB2 gene. The vertical bars locate the binding sites. **D.** Methylation analysis of CpG island 2 of the MAGEB2 gene in mTECs, treated or not with the AIRE-specific siRNA SI0211352, and in B16F10 melanoma cells. DNA was predigested with FNU4H1 restriction enzyme, which does not degrade methylated DNA. Then, specific primers were used to selectively amplify the CpG 2 island by PCR. Agarose gel electrophoresis shows the presence of a gene amplification product (350 pb) in the columns corresponding to undigested DNA from mTECs (c) or digested DNA from mTECs pretreated with the AIRE-specific siRNA (d), but not from untreated mTECs (b) or B16F10 cells (a). The results of one representative experiment out of two are shown.

To test whether the two allelic variants of mouse AIRE gene induce different levels of MA expression in mTECs, AIRE (GeneBank accession number: AF073797) and MAGEB2 (GeneBank accession number: AC097273.3) gene expressions were comparatively studied in mTECs from C57BL/6 mice strain 1 and 2. Figure 2A shows that relative AIRE and MAGEB2 gene expressions were significantly higher in mTECs from strain 1 than in those from strain 2 mice, and that AIRE gene expression in mTECs from each of the two strains could be efficiently silenced by both siRNAs SI0211352 and S100186424. Importantly, AIRE gene silencing also abolished the expression of MAGEB2 gene but not that of the AIRE non-dependent GAD1 gene (GeneBank accession number: AC097273.3). In these experiments AIRE gene silencing was effective since AIRE protein was detectable in untreated mTECs from both strains of C57BL/6 mice, but neither in the same cells treated with the two AIRE-specific siRNA nor in RMA-S control cells (Figure 2B).

Gene sequence analysis of MAGEB2 and relative proximal gene regions by the Pattern matching/DNA-pattern tools (http://rsat.ulb.ac.be/rsat) revealed six potential AIRE-binding sites (T-boxes and G-boxes) [24] located from 0.5 kb to approximately 10 kb upstream ATG initiation codon of MAGEB2 gene (Figure 2C). Since the expression of MAGE genes is regulated by DNA demethylation [25], and epigenetic control of methylation is one of AIRE mechanisms of action [26], the methylation status of the CpG islands upstream the MAGEB2 gene in mTECs treated or not with an AIRE-specific siRNA was evaluated. Three CpG islands (1, 2, 3) located 59.794, 474.936, and 678.816 kilobases upstream the MAGEB2 initiation codon (UCSC database), respectively, were analyzed. In order to selectively amplify methylated genomic regions, mTECs DNA was pre-digested with FNU4H1 enzyme, which degrades only demethylated DNA. Interestingly, a band corresponding to CpG island 2 of MAGEB2 gene was observed after PCR amplification of DNA from mTECs pre-treated with an AIRE-specific siRNA but not from untreated mTECs or B16F10 melanoma cells, which costitutively express MAGEB2 (Figure 2D). No differences were detected concerning CpG islands 1 and 3 (not shown).

### Identification of MAGEB2-derived immunogenic peptides

MAGEB2_256-264_ (SDPPSYEFL) and MAGEB2_134-142_ (KYKEQFPEI) peptides were selected as potential high and low H-2 K^b^ binders, respectively, based on their relative binding scores (66.000 vs 0.720, respectively), using the BIMAS epitope prediction software (www-bimas.cit.nih.gov).

**Figure 3:**
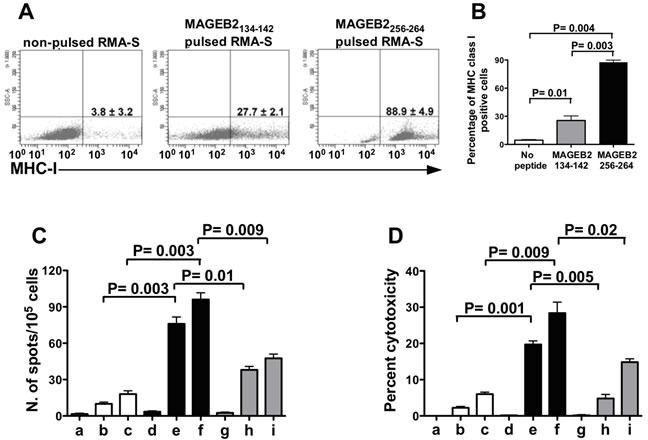
Frequency and cytotoxic activity of MAGEB2_**256-264**_ peptide-specific CD8+ T cells **A.** MHC-I stabilization assay performed pulsing RMA-S cells with either the MAGEB2_134-142_ or the MAGEB2_256-264_ peptide. The results refer to one out of three experiments and are expressed as percentages of positive cells. The mean values ± SD of the three experiments and relative statistically significant differences, calculated by paired t test, are reported in the histogram. (**B**) Frequency of IFNγ secreting T cells and (**C**) cytotoxic activity (**D**) of different CD8+ T cell populations incubated in the presence of either RMA-S cells, pulsed or not with MAGEB2 peptides, or B16F10 melanoma cells. Unstimulated CD8+ T splenocytes (□), CD8+ T splenocytes cultured for 5 days in the presence of syngeneic DC pulsed with MAGEB2_256-264_ peptide (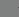) and CD8+ T splenocytes cultured for 5 days in the presence of syngeneic DC pulsed with MAGEB2_134-142_ peptide (■) were used as effector cells. Non-pulsed RMA-S (a, d, g), RMA-S pulsed with MAGEB2_256-264_ peptide (b, e), RMA-S pulsed with MAGEB2_134-142_ peptide (h) and B16F10 melanoma cells (c, f, i) were used as targets. Effector:target ratio was 10:1 for (C) and 20:1 for (D). The figures show the results of one out of three experiments. Results are expressed as mean values of duplicate wells. The statistically significant differences of frequencies or cytotoxic activity among different CD8+ T cell populations, calculated by unpaired t test, are indicated.

To validate the results of this analysis, the MHC class I antigen stabilization assay was performed using TAP-deficient RMA-S cells, which express surface MHC class I antigen only when pulsed with an exogenous peptide. In this stabilization assay, the percentage of RMA-S cells expressing MHC class I antigens was 88% when the cells were pulsed with the MAGEB2_256-264_ peptide, while it was 27% following pulsing with the MAGEB2_134-142_ peptide (Figure 3A).

Then, the immunogenicity of these peptides was analyzed by Elispot and by cytotoxic assay. In these assays C57BL/6 mouse splenocytes were stimulated with syngeneic dendritic cells (DC) pulsed either with the MAGEB2_256-264_ or the MAGEB2_134-142_ peptide. The results of these assays showed that the stimulation with the MAGEB2_256-264_ peptide induced a higher frequency of IFNγ secreting cells and a higher cytotoxic activity than stimulation with the MAGEB2_134-142_ peptide (Figures 3B and 3C). Hence, the MAGEB2_256-264_ peptide showed to be more immunogenic than MAGEB2_134-142_ peptide.

### AIRE rs1800522 SNP modulates the apoptotic rate in MAGEB2-specific cytotoxic T lymphocytes (CTL) exposed to mTECs

**Figure 4:**
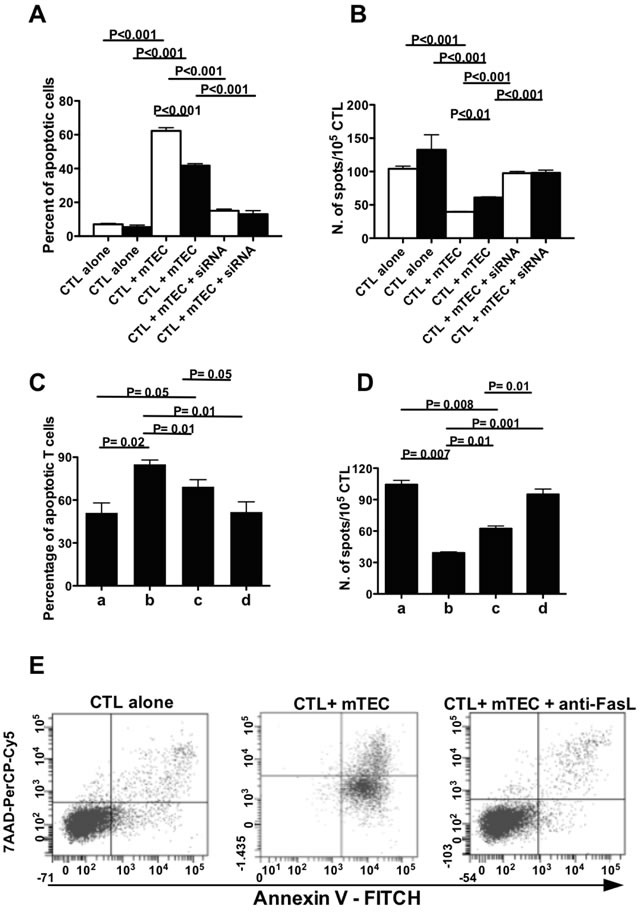
rs1800522 SNP regulates MAGEB2 expression and MAGEB2-specific T cell survival **A.** Percentage of apoptotic MAGEB2_256-264_ specific CTL cultured for 24 h alone or with mTECs from either C57BL/6 strain 1 (white columns) or 2 (black columns) mice untreated or treated with the AIRE-specific siRNA SI0211352. The data are the mean of 3 independent experiments. Each experiment was performed in triplicates. **B.** Frequency of IFNγ secreting cells in MAGEB2_256-264_ specific CTL cultured for 24 h alone or with mTECs from either C57BL/6 strain 1 (white columns) or 2 (black columns) mice untreated or treated with the AIRE-specific siRNA SI0211352. The data are the mean of 3 independent experiments. Each experiment was performed in triplicates. **C.** Percentage of apoptotic MAGEB2_256-264_ specific CTL cultured for 24 h in culture medium alone (a) or in the presence of wild type mTECs (bearing the TT genotype) (b), transgenic mTECs (bearing the CC genotype) (c), AIRE silenced mTECs (d). The data are the mean of 3 independent experiments. Each experiment was performed in triplicates. **D.** Frequency of IFNγ secreting MAGEB2_256-264_ specific CTL cultured for 24 h alone (a) or in the presence of wild type mTECs (bearing the TT genotype) (b), transgenic mTECs (bearing the CC genotype) (c), AIRE silenced mTECs (d). The data are the mean of 3 independent experiments. Each experiment was performed in triplicates. **E.** Percentage of apoptotic MAGEB2_256-264_ specific CTL cultured for 24 h alone or with mTECs from C57BL/6 strain 1 in the presence or not of an anti-FasL mAb (0.5 μg/ml). A gate was established on CD3+CD8+ T lymphocytes. The dot plots show the percentages of apoptotic CD3+CD8+ T cells (positive for annexin V staining and negative for 7AAD staining) in the different culture conditions. An isotype-matched mAb was used as a control: under these culture conditions the percentage of apoptotic CD3+CD8+ T cells was comparable to that of CD3+CD8+ T cells cultured with mTECs in the absence of the antibody (not shown). The results of one representative experiment out of three are shown.

MAGEB2_256-264_ peptide-specific CTL from strain 1, cultured in the presence of syngeneic mTECs, showed a higher extent of apoptosis (Figure 4A and Supplementary Figure 1) and a reduced frequency (Figure 4B) than corresponding CTL from strain 2 mice cultured with syngeneic mTECs. In the presence of a neutralizing anti-Fas ligand mAb the extent of apoptosis of MAGEB2_256-264_ peptide-specific CTL co-cultured with syngeneic mTECs was comparable to that of CTL cultured alone (Figure 4E), suggesting that a Fas-dependent mechanism was involved. Hence, the TT AIRE genotype, present in C57BL/6 mice strain 1, is associated with higher MAGEB2 gene expression and greater extent of apoptosis of MAGEB2-specific CTL than the corresponding CC genotype. In agreement with these observations, spectratyping of the T cell receptor (TCR) complementary determining region (CDR) 3 of MAGEB2_256-264_-specific CTL, showed different T cell repertoires when the cells were cultured alone, in the presence of untreated mTECs or in the presence of AIRE silenced mTECs, supporting the direct role played by AIRE in modulating the T cell repertoire (Supplementary Figure 2).

To confirm these data, mTECs from strain 1 were made transgenic for the C allelic variant (Supplementary Figure 3A) and the relative functions were analyzed. AIRE and MAGEB2 genes displayed a significantly lower expression in mTECs bearing the CC transgenic genotype than in non-transgenic mTECs, and were efficiently silenced by an AIRE-specific siRNA (Supplementary Figure 3B). Non-transgenic mTECs, co-cultured with syngeneic MAGEB2_256-264_ peptide-specific CTL, induced a higher extent of apoptosis (Figure 4C) and a lower frequency of MAGEB2_256-264_ peptide-specific CTL (Figure 4D) than transgenic mTECs.

### B16 melanoma tumor growth in C57BL/6 mice strain 1 and 2 immunized against MAGEB2

**Figure 5:**
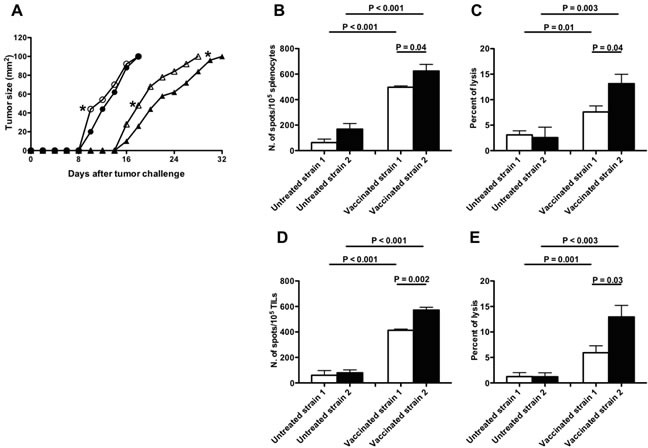
Effects of MAGEB2 vaccination in B16F10 melanoma-challenged C57BL/6 strain 1 and 2 mice **A.** Melanoma growth curves. Growth curves of B16F10 melanoma in C57BL/6 strain 1 (white symbols) and 2 (black symbols) mice vaccinated (triangles) or not (circles) with syngeneic MAGEB2_256-264_ peptide-pulsed DC. Unpaired t test analyses were performed to verify the existence of statistically significant differences between untreated strain 1 and strain 2 groups or between vaccinated strain 1 and strain 2 groups in day-times needed by the tumor to reach either 40 mm^2^ or 100 mm^2^ dimensions. *: *P* < 0.05. Similar analyses comparing untreated with vaccinated strain 1 or untreated with vaccinated strain 2 groups showed the existence of highly significance statistical differences (*P* < 0.0001). **B.**-**E.** Frequency and cytotoxic activity of MAGEB2_256-264_ peptide-specific CD8+ T cells from strain 1 and strain 2 mice after MAGEB2 immunization and tumor challenge. (B) and (C) panels refer to analyses performed on splenocytes; (D) and (E) panels refer to analyses performed on Tumor Infiltrating Lymphocytes (TIL). In all panels white bars indicate analyses executed on C57BL/6 strain 1 mice, while black bars indicate analyses executed on C57BL/6 strain 2 mice. (B) and (D) panels show the frequency of T cells specifically secreting IFNγ after incubation with MAGEB2_256-264_ peptide-pulsed RMA-S cells (Elispot analyses); the effector:target ratio was 10:1. Non-pulsed RMA-S cells were used as negative control: the number of spots in wells containing effector cells and non-pulsed RMA-S cells was considered as non-specific background. Data are presented as: frequency of spots in cultures of effector cells plus MAGEB2_256-264_ peptide-pulsed RMA-S cells - frequency of spots in cultures of effector cells plus non-pulsed RMA-S cells. (C) and (E) panels show the cytotoxic activity exerted by effector lymphocytes (splenocytes or TIL) against MAGEB2_256-264_ peptide-pulsed RMA-S cells (cytotoxic assay); the effector:target ratio was 20:1. Cultures of effector cells plus non-pulsed RMA-S cells were used as negative control.

To assess the *in vivo* relevance of AIRE functional differences related to rs1800522 SNP, B16F10 melanoma tumor growth was comparatively measured in mice from strain 1 and 2. Tumor growth was significantly (p<0.05) lower in strain 2 than in strain 1 mice until the 10^th^ day from tumor challenge, but not at later stages (Figure 5A). However, when both strains were immunized before tumor challenge with syngeneic DC pulsed with MAGEB2_256-264_ peptide, melanoma tumor growth was significantly (p<0.05) lower in strain 2 than in strain 1 mice at all time-points (Figure 5A). Interestingly, higher frequency of MAGEB2_256-264_ peptide-specific IFNγ secreting T cells and higher CD8^+^ T cell cytotoxic activity were observed in strain 2 than in strain 1 mice (Figures 5B, 5C, 5D, 5E).

## DISCUSSION

The results of our study show that: a) AIRE controls MAGEB2 expression by mTECs through an epigenetic mechanism; b) variant alleles of the rs1800522 SNP of human AIRE gene differently regulate MAGEB2 expression by mTECs; c) mTECs expressing different MAGEB2 levels under the control of variant alleles of AIRE induce different extent of apoptosis in MAGEB2-specific CTL; and d) the C allelic variant of AIRE rs1800522 SNP (protective in humans) is associated with a more effective MAGEB2-specific T cell immune response than the corresponding T allelic variant.

Human MAGEB2 antigen displays a high degree of homology with its mouse counterpart (BLAT analysis by UCSC genome browser) and is associated with a T cell-specific protective immune response [27]. We show here that AIRE regulates MAGEB2 gene expression through gene demethylation, suggesting that the modelling of MAGEB2-specific T cell repertoire is under the direct control of AIRE. On this basis, variations of AIRE gene activity, due to polymorphisms or gene alterations, could be related to protection or susceptibility to melanoma, as supported by several lines of evidence in humans [15] and in animals [28]. Interestingly, we observed that the C variant allele of the AIRE gene sequence corresponding in mice to human rs1800522 SNP induced lower MAGEB2 gene expression in mTECs and, subsequently, lower MAGEB2-specific, Fas-dependent CTL apoptosis than the C variant allele present in mTECs of strain 2 mice. Accordingly, strain 2 mice, bearing the AIRE CC genotype, protective in humans [15], showed in the periphery higher frequency of MAGEB2-specific T cells and higher MAGEB2-specific T cell cytotoxicity than strain 1 mice bearing the AIRE TT genotype. Indeed, vaccination with MAGEB2_256-264_ -peptide had a higher protective efficiency against B16F10 melanoma tumor growth in strain 2 than in strain 1 mice. Hence, for the first time our data elucidate, at the genetic and molecular level, the processes linking the expression of a specific AIRE gene polymorphism to susceptibility to a neoplastic disease such as melanoma. This finding allows one to envisage the possibility to map single individuals in order to predict genetic predispositions to tumor development, which could be counteracted by adoption of opportune preventive interventions or by specific immunotherapy.

## MATERIALS AND METHODS

### Mice

Six-12 week old C57BL/6 female mice (H-2 K^b^/D^b^) were purchased from Harlan Srl (Indianapolis, IN) and from Charles River (Wilmington, MA) between January 2011 and December 2014. The protocol here adopted received approval by the Ethics Committee for Experimentation on Animals of IRCCS Azienda Ospedaliera Universitaria San Martino-IST of Genoa.

### Cell lines

RMA-S MHC class I antigen negative mutant mouse lymphoma cells [29] were a kind gift of Dr. G. Pietra (Advanced Biotechnology Center, Genoa) while B16F10 melanoma cells (syngeneic to C57BL/6 mice) were purchased from ATCC. Both cell lines were received in 2012: since then, the cell lines were tested at six-month intervals. In particular, RMA-S cells were analyzed for the deficient expression of MHC class I antigens using the MHC class I-specific mAb clone 28-8-6 (PE, Biolegend, San Diego, CA); B16F10 cells were analyzed by RT-PCR and gene sequencing on amplified cDNA for MAGEB2 MA gene expression. RMA-S and B16F10 cell lines were grown at 37 C in a 5% CO_2_ atmosphere in RPMI-1640 medium (SIGMA, St. Louis, USA) supplemented with 10% FCS, 2 mM L-glutamine, 10 mmol HEPES, 100 mmol non-essential amino acids, 100 U/ml penicillin, 100 μg/ml streptomycine, and 1% sodium pyruvate (referred to as culture medium).

### T cell sorting

TILs were purified from melanoma tumors filtering minced tissues using sterile cell strainers (Falcon, BD Biosciences, San Josè, CA). Collected cells were centrifuged on Ficoll gradient. Total T lymphocytes were sorted from TIL using Dynabeads® FlowComp™ Mouse Pan T (CD90.2) isolation kit (Invitrogen by Life Technologies Ltd., Paisley, UK) following the manufacturer's instructions. The purity of total T lymphocyte preparations was monitored by flow cytometric analysis of cells stained with the allophycocianin (APC)-cyanin (Cy) 7 conjugated anti-CD8 monoclonal antibody (mAb) clone 53-6.7 and the Peridinin Chlorophyll Protein Complex-cyanin 5.5 (PerCP-Cy5.5) anti-CD3 mAb clone 145-2C11 (BD Bioscience, New Jersey, USA).

### Generation of mTEC cultures

mTECs were isolated as previously described [30]. They were cultured in DMEM medium supplemented with D-valine (94 μg/ml), glutathione (0.1 mg/ml), ascorbic acid (50 μg/ml), insulin (0.4 μg/ml), L-glutamine (0.58 mg/ml), 10% FCS and 1% Pen/Strep antibiotics in 6 well-plates. Cells were phenotypically characterized by flow cytometry using the EpCAM-specific monoclonal antibody (mAb) G8.8 (Biolegend, San Diego, CA) and the MHC class-II antigen-specific mAb clone 25-9-17 (BD Biosciences, Franklin Lakes, NJ).

### Expression of AIRE, MAGEB2 and Glutamate decarboxylase 1 (GAD1) genes in mTECs

AIRE, MAGEB2 and GAD1 gene expression was analyzed by PCR and real time PCR as previously described [31]. Briefly, 48 μl of RNA, isolated using the OMNIZOL RNA Isolation kit (EuroClone, London, UK), were treated with 6 U DNase I and reverse transcribed into cDNA using Oligo(dT) 20Primer and Superscript II Reverse Transcriptase, followed by RNase H digestion. The amplification program included: a single denaturation step at 94°C for 3 min followed by either 35 (for MAGEB2), or 30 (for AIRE and for β-actin) cycles at 94°C for 1 min, then 54–62°C for 1 min and 72°C for 2 min, followed by a final extension step at 72°C for 10 min. The following sense and antisense, respectively, oligonucleotide pairs were used: AIRE, 5′-GCAACTCTGGCCTCAAAGAG-3′and 5′-GGTCTGAATTCCGTTTCCAA-3′; MAGEB2, 5′-GAGCTTGATCCACGAGTT-3′ and 5′-AGGAGACCTGTCCTGTCCTAGGC-3′; GAD1, 5′-ATAGAAAGGGCCAATTCAGTC-3′, and 5′ -TGCATCCTTGGAGTATACCC-3′; β-act, 5′- TCATGAAGTGTGACGTTGACATCCGT-3′ and 5′-GTCAGGATCTTCATGAGGTAGTC-3′.In order to obtain quantitative measures of AIRE and MAGEB2 gene expression, quantitative real time PCR was performed using the LightCycler thermocycler and the SYBR Green Master Mix (Roche Diagnostics, Mannheim, Germany). The same primers were used for the real time PCR. AIRE and MAGEB2 PCR products were confirmed by sequence analysis.

### AIRE gene silencing

SI0211352 and S100186424 AIRE-specific siRNA (Qiagen, Hilden, Germany) were used for silencing AIRE gene expression in mTECs. The sequences of sense and antisense strands of AIRE-specific siRNA were respectively: 5′-GGAAGAUCCAAGAAGUGCATT-3′ and 5′-UGCACUUCUUGGAUCUUUCCTG-3′ for SI0211352; 5′-GUGGCAAUUUGAAGAACAATT3′ and 5′-UUGUUCUUCAAAUUGCCACTG-3′ for S100186424. mTECs (4×10^5^) were transfected with 2.5 μg of AIRE-specific siRNA using the liposomal transfection reagent (Roche, Mannheim, Germany). Cells were cultured for 24 h at 37°C in culture medium before extracting total RNA and quantifying AIRE mRNA expression by Real Time PCR.

### AIRE gene product detection by western blot

mTECs were pelleted in cold lysis buffer containing 50 mM Tris-HCl pH 8.0, 150 mM NaCl, 1% NP-40, and a protease inhibitor cocktail (G-Bioscience, St. Louis, MO). Protein concentration was determined by the Bio-Rad Protein Assay (Bio-Rad Laboratories Inc, Hercules, CA) and AIRE protein was detected by western blot [32] using AIRE-specific rabbit antibodies (1:1000, Millipore, Billerica, MA), peroxidase-conjugated goat anti-rabbit IgG antibodies (1:2000, Millipore, Billerica, MA) and the Western Blotting Luminol Reagent (Roth, Karlsruhe, Germany).

### Generation of transgenic mTECs expressing the CC AIRE genotype at the rs1800522 SNP site

A plasmid shuttle vector bearing the CC genotype of rs1800522 SNP was prepared as previously described [24]. Briefly, we cloned in the SacII unique cloning site of pcDNA3.1/V5-His-TOPO plasmid (Invitrogen, Carlsbad, CA) a 400 pb AIRE gene sequence. To this aim, a bacterial artificial chromosome containing the C57BL/6 AIRE gene sequence (RPCI23.C 278E6 clone, Life Technologies, Paisley, UK), selected according to UCSC database, was used as template and in situ-direct mutagenesis of the wild-type TT genotype was performed using AIRE 5′-tccccgcggtggaaaaactgaggaccttctcca-3′ and 5′-tccccgcggcaggagtataccccccgaaa-3′ external primers as well as 5′-tcagcactcatttgacggcatcctg-3′ and 5′-caggatgccgcaaatgagtgctga-3′ internal primers designed on the polymorphic sequence. Plasmid sequence was then analysed to monitor the AIRE genotype substitution.

This plasmid, containing the G418 resistance gene, was used as a partner in homologous recombination [33]. mTECs (5×10^5^) were transfected overnight with 2 μg of plasmid using a Polifect transfection kit (Qiagen, Hilden, Germany) and selected and expanded in medium supplemented with G418 (1 mg/ml). Total cellular DNA from growing colonies was sequenced to confirm that recombination had occurred.

### Identification of AIRE-binding sites on MAGEB2 promoter gene

The Pattern matching/DNA-pattern tools (http://rsat.ulb.ac.be/rsat) was used to predict AIRE transcription factor binding sites (T-boxes and G-boxes) located from 0.5 kb to 10 kb upstream ATG initiation codon of MAGEB2 gene [24].

### CpG methylation pattern analysis of MAGEB2 upstream promoter sequences

DNA was digested with FNU4H1 (Biolabs, Ipswich, MA), a restriction enzyme sensitive to methylation. Digested and undigested DNA were used as templates for PCR performed with specific primers designed on CpG islands of MAGEB2 gene sequence selected according to UCSC genome browser database. The following specific primers were selected according to UCSC genome browser database: CpG 1, 5′-CGTCCCCCTCTGCGCCT-3′ and 5′-TTCCCGAGACACGTGCGTTTGCTGA-3′; CpG 2, 5′-GGCTGGTCTCCGGGGTGA-3′ and 5′-CTTCGTCAGCTGGTCGCACCC-3′; CpG 3 5′-GCAGAACCCACGGTCTCGC-3′ and 5′-GATCAAGCCCCTCCACCCCCA-3′. PCR products were then sequenced.

### Gene sequencing

Gene sequencing was performed as described [34]. Briefly, DNA sequencing was carried out using an ABI 3130XL automated sequencer (Applied Biosystems, Foster City, CA). DNA sequence analyses were performed on PCR products amplified using forward primers specific for AIRE (5′-TGACTTCTTGTAACGATG-3′), MAGEB2 (5′-GAGCTTGATCCACGAGTT-3′), or for CpG island sequences (CpG1: 5′-CGTCCCCCTCTGCGCCCT-3′, CpG2: 5′-GGCTGGTCTCCGGGGTGA-3′, CpG3: 5′-GCAGAACCCACGGTCTCGC-3′).

### Epitope prediction and peptide synthesis

Potential H-2 K^b^ binder 9mer peptides from MAGEB2 murine protein were identified using the BIMAS epitope prediction software (www.bimas.cit.nih.gov). Peptides were synthesized using the standard method of solid-phase peptide synthesis, which follows the 9-fluorenylmethoxycarbonyl (Fmoc) strategy with minor modifications [35, 36].

### MHC class I antigen stabilization assay

MHC class I antigen stabilization assay was performed as described [37, 38]. Briefly, RMA-S cells were cultured at 26°C overnight and washed with PBS. Then, cells (1×10^6^ cells/ml in RPMI-1640 medium) were incubated at 37°C for 1 hour with either MAGEB2_256-264_ (50 μg/ml) or MAGEB2_134-142_ peptides (50 μg/ml). After washings, cells were stained with the labelled MHC class I antigen-specific mAb clone 28-8-6 (PE, Biolegend, San Diego, CA) and analyzed with a FACSCanto cytometer (BD Biosciences, New Jersey, USA).

### Splenic CD8^+^ T cell purification

CD8^+^ splenic T cells were enriched by immunomagnetic cell sorting using the MACS system (Milteny Biotec, Bergisch Gladbach, Germany). CD8^+^ T cell enrichment (> 95%) was monitored by FACS analysis using the FITC-conjugated CD8-specific mAb clone 53-6.7 and the APC-Cy7 labelled CD3-specific mAb clone 145-2C11 (BD Bioscience, Franklin Lakes, NJ).

### Generation of DC from murine bone marrow cells

DC were generated as described [39]. Briefly, bone marrow was flushed from the long bones of the hind limbs of 10-14 week old female C57BL/6 mice. The cells were cultured in DC medium consisting in RPMI supplemented with 10%FCS, 2 mmol/l L-glutamine, 10 mmol HEPES, 100 mmol non-essential amino acids, 100 U/ml penicillin, 100 μg/ml streptomycine, 50 μmol β- mercaptoethanol, 20 ng/ml GM-CSF (Sigma-Aldrich, St. Louis, USA). On day 6 or 7 of culture, released mature non-adherent cells with the typical features of DC were used for *in vitro* studies.

DC were phenotypically analyzed using CD11c-specific mAb clone 2D7, PE-Cy7, CD86-specific mAb clone GL1, APC, MHC class II antigen—specific mAb clone 25-5-17, FITC (Biolegend, San Diego, CA): >90% of cells showed high expression of these markers.

### MAGEB2-specific CTL generation

DC (2×10^6^) were pulsed with either MAGEB2_256-264_ or MAGEB2_134-142_ peptide at 10 ng/ml for 1 hour at 37°C. Peptide-pulsed DC (5×10^4^ cells/well) were incubated for 10 days at 37°C with purified splenic CD8^+^ T cells (5×10^5^ cells/well) seeded in 24-well plates. IL-2 Recombinant Mouse Protein (50 IU/ml) (Invitrogen, Carlsbad, CA) and MAGEB2_256-264_ peptide (70 ng/ml) were added to the cultures on days 1, 4 and 7. Cells were then expanded by repeated stimulations with peptide-pulsed DC (1×10^3^ cells/well) and IL-2 (50 IU/ml).

### Enzyme-linked immunospot assay (ELISPOT)

ELISPOT was performed using the INF-γ enzyme-linked immunospot antibody Pair, Mouse (Millipore, *Billerica*, MA) following the manufacturer's instructions. Responder cells (TILs isolated from melanoma tumors, CD8^+^ T cells isolated from fresh splenocytes, MAGEB2_256-264_ or MAGEB2_134-142_ peptide-specific CD8^+^ T cells) (1×10^5^/well) were incubated with homologous peptide-pulsed RMA-S cells (1×10^4^/well) or B16F10 cells (1×10^4^/well) in a final volume of 100 μl. Spots were counted using a BioReader 3000 Elispot Reader (Bio-Sys GmbH).

### Cytotoxic assay

The assay was performed as described [40]. Briefly, RMA-S cells (5×10^3^ cells/well) pulsed with the MAGEB2_256-264_ peptide, or B16F10 cells (5×10^3^ cells/well) were stained with carboxyfluorescein diacetate succinimidyl ester (CFSE) and cultured with MAGEB2-specific CTL effector cells (1×10^5^ cells/well), or freshly purified CD8+ splenocytes (1×10^5^ cells/well) as negative control. After 4 hour incubation at 37°C cells were stained with 7-Amino-actinomycin D (7-AAD). Spontaneous target cell lysis and lysis of non-pulsed RMA-S cells were considered as background. The mean percentage of specific lysis was calculated as follows: % specific lysis =[(viable target cells of control sample-viable target cells of test sample)/ viable target cells of control sample] x 100. The assay was performed in duplicate.

### TCR CDR3 spectratyping

MAGEB2_256-264_-specific CTL (1×10^6^ cells/well) from strain 1 mice were cultured for 24 h alone or co-cultured with either untreated syngeneic mTEC (5×10^5^ cells/well) or with syngeneic mTEC (5×10^5^ cells/well) silenced for the expression of AIRE gene as above described. TCR CDR3 length analysis, also referred as Spectratyping or Immunoscope, was performed as follows. cDNAs recovered from the different samples and culture conditions were amplified under non-saturating PCR conditions with TCR-Vβ family-specific primers, as described [41–43]. Each different PCR product was run in a fluorescence-based DNA sequencer (ABI 377; Applied Biosystems) with Rox-labeled size markers (Applied Biosystems). The data were analyzed using Genescan software (Applied Biosystems) to assign size and peak areas to the different PCR products.

### Analysis of CTL apoptosis and inhibition of Fas-mediated apoptosis

CTL apoptosis was analyzed incubating MAGEB2_256-264_-specific CTL (1×10^5^ cells/well) for 24h at 37°C with mTECs (1×10^5^ cells/well) expressing either the TT or the CC genotype of AIRE gene in 100 μl final volume of culture medium. Non-adherent cells were, then, harvested, viable cells counted by 7AAD exclusion, and stained with FITC annexin V kit as well as CD8-specific APC-mAb (Biolegend). FACS analysis was then performed using the FACSCanto flow cytometer. A blocking hamster IgG anti-mouse Fas ligand mAb (clone MFL3, eBioscience, San Diego, CA) was used to study the dependency of mTEC-induced CTL apoptosis on Fas-Fas ligand interaction. To this aim, mTEC were co-cultured with CD8^+^MAGEB2_256-264_ specific T cells for 24h in the absence or the presence of a hamster IgG anti-mouse Fas ligand mAb (clone MFL3, Ebioscience, New Jersey, USA), blocking Fas-mediated cell death (used at either 0.5 or 2.5 μg/ml concentrations). A unrelated, isotype-matched mAb (clone MAR-1, Ebioscience, New Jersey, USA) was used as control. The analysis of apoptosis on CD8^+^MAGEB2_256-264_-specific T cells was performed as above described. The experiments were repeated 3 times in duplicate.

### Vaccination with MAGEB2256-264 loaded DC of C57BL/6 mice bearing TT or CC AIRE genotype

Before being challenged with B16F10 melanoma cells, both C57BL/6 strain 1 and strain 2 mice were immunized with MAGEB2_256-264_ peptide-pulsed DC, as follows. Syngeneic DC (2×10^6^ cells), pulsed with MAGEB2_256-264_ peptide (50 μg) for 1 h at 37°C, were intradermally injected at the base of the tail of each mouse. Immunizations were performed on days −21, −14 and 0 with respect to tumor challenge. The day of the third immunization, B16F10 melanoma cells (1×10^5^/0.1 ml of sterile PBS) were intradermally injected. The kinetics of melanoma growth was monitored by measurements with a microcaliper every other day. Mice were sacrificed when palpable tumors reached 10 mm of maximum diameter. Each sample group of animals included 6 mice; the experiments were repeated twice.

### Statistical analyses

The statistical significance of differences between means was analyzed using the Mann-Whitney *t* test for non-parametric values or the paired t test for paired measures. Calculations were performed using GraphPad Prism version 5.0 software: two sided tests were performed. The relative gene expression levels were calculated using the comparative *C*_t_(ΔΔ_t_) method [44].

## SUPPLEMENTARY MATERIALS FIGURES


