# Supplementary material for: AIRE polymorphism, melanoma antigen-specific T cell immunity, and susceptibility to melanoma
Source: Oncotarget. 2016 Aug 22;7(38):60872–84. doi: 10.18632/oncotarget.11506 (PMC5308622; doi:10.18632/oncotarget.11506)
Supplement: Supplementary file 1 [file oncotarget-07-60872-s001.pdf]

AIRE polymorphism, melanoma antigen-specific T cell immunity, and susceptibility to melanoma

Supplementary Material

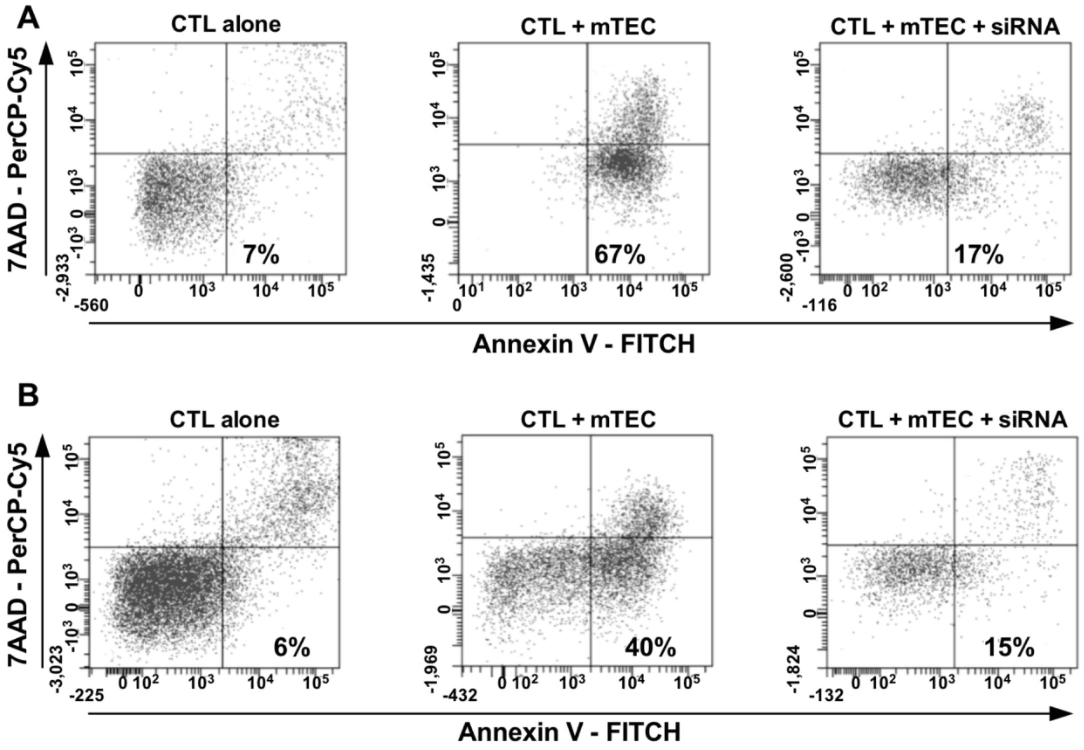

Supplementary Figure 1. Representative experiment showing the percentage of apoptotic MAGEB2$_{256\text{-}264}$ specific CTL cultured alone or with mTECs from either C57BL/6 strain 1 (**A**) or 2 (**B**) mice. Left panels refer to CTL cultured in culture alone, middle panels refer to CTL-mTECs co-cultures, right panels refer to CTL-mTEC co-cultures performed after silencing AIRE gene expression in mTECs by a specific siRNA. The percentages of apoptotic CTL are indicated in each panel.

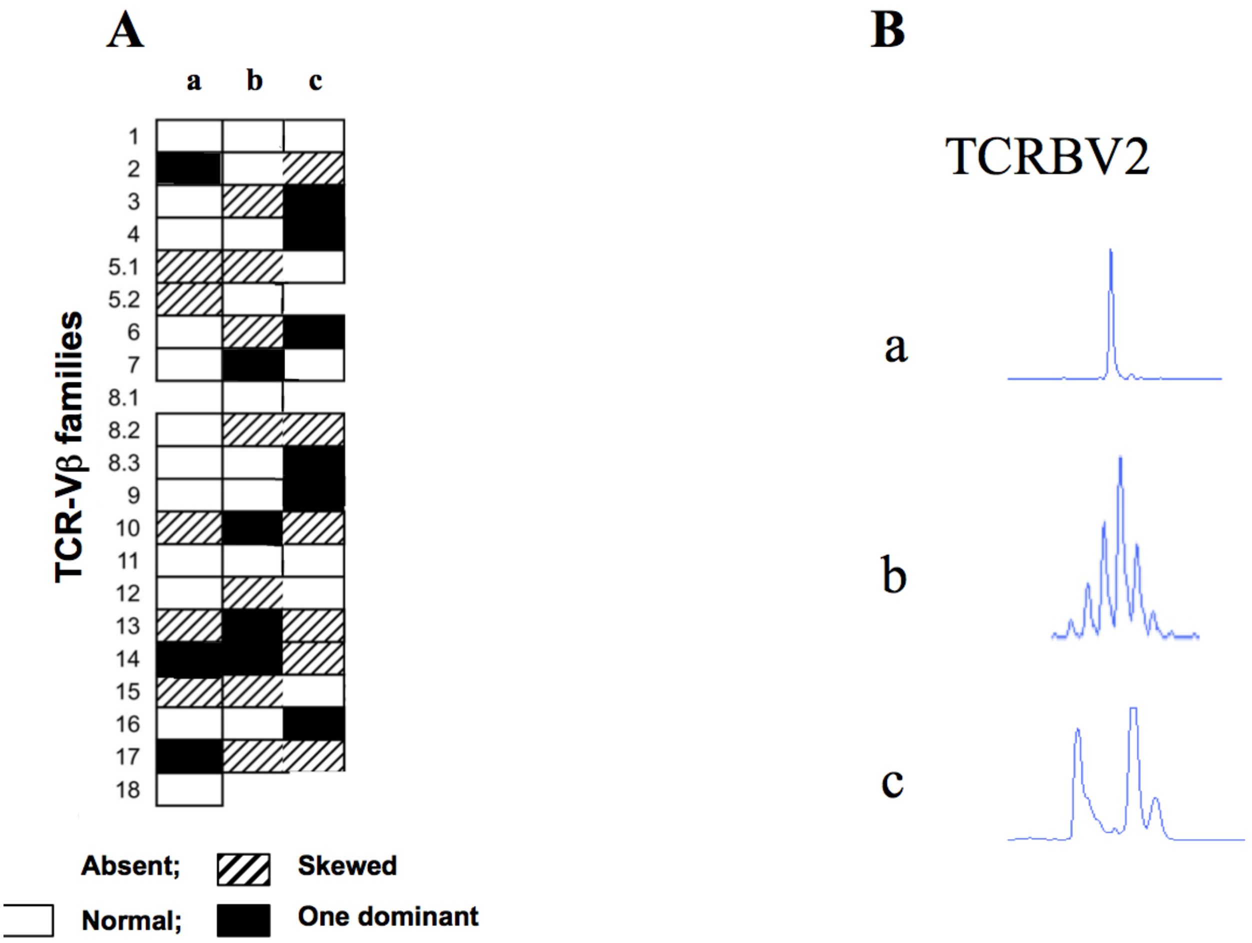

Supplementary Figure 2. TCR CDR3 spectratyping of a MAGEB2$_{256\text{-}264}$ specific CTL. A MAGEB2$_{256\text{-}264}$ specific CTL was cultured alone (**a**), in the presence of syngeneic mTECs (**b**), or in the presence of syngeneic mTECs in which AIRE gene expression had been silenced by the SI0211352 AIRE-specific siRNA (**c**). **A**) Synoptic table of the entire TCR-Vβ family repertoire. Here, the single TCR-Vβ family is shown as a non-closed box when the signal for the given family is not found (Absent), a diagonal stripe pattern box when there is a skewed repertoire (Skewed), a black box when a single predominant peak is found (One dominant), and a white box when the family has a normal profile (Normal). **B**) Representative profile of the TCR Vβ2 family.

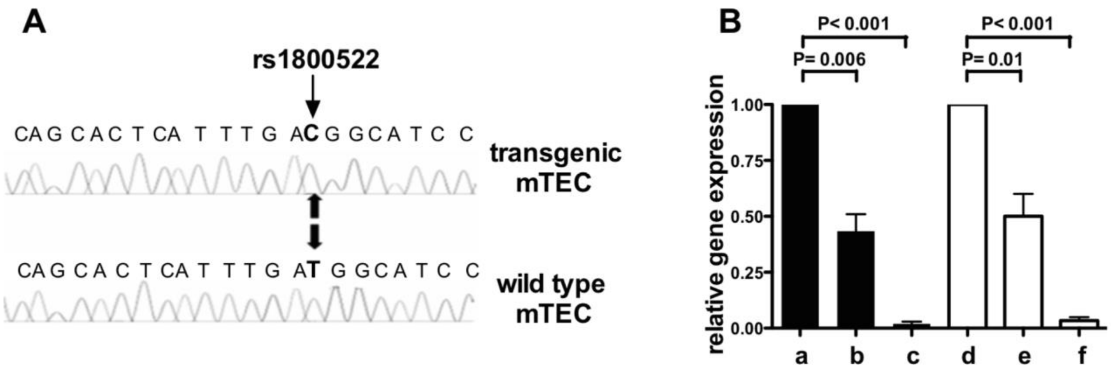

Supplementary Figure 3. Transgenesis of mTECs from C57BL/6 strain 1 mice with the C allelic gene variant from strain 2 mice. (**A**) AIRE gene sequences of the variant alleles for rs1800522 SNP. DNA extracted from thymus purified (wild type) mTECs or from mTECs made transgenic for the C allelic variant was amplified by primers specific for the exon 14 of AIRE gene, which encompasses the rs1800522 SNP. Subsequently, PCR products were sequenced. The T→C nucleotide substitution at the site corresponding to the rs1800522 SNP is indicated by divergent arrows. (**B**) AIRE (black columns) and MAGEB2 (white columns) gene expression in: (a) and (d) thymus purified mTECs, (b) and (e) transgenic mTECs, (c) and (f) thymus purified mTECs treated with an AIRE-specific siRNA.
